# Genome-wide analysis of basic helix-loop-helix (bHLH) transcription factors in *Brachypodium distachyon*

**DOI:** 10.1186/s12864-017-4044-4

**Published:** 2017-08-15

**Authors:** Xin Niu, Yuxiang Guan, Shoukun Chen, Haifeng Li

**Affiliations:** 1 State Key Laboratory of Crop Stress Biology for Arid Areas, College of Agronomy, Northwest A&F University, Yangling, China; 2 Xinjiang Agricultural Vocational Technical College, Changji, China

**Keywords:** Genome-wide, bHLH, *Brachypodium distachyon*, Expression profile, Transcription factor

## Abstract

**Background:**

As a superfamily of transcription factors (TFs), the basic helix-loop-helix (bHLH) proteins have been characterized functionally in many plants with a vital role in the regulation of diverse biological processes including growth, development, response to various stresses, and so on. However, no systemic analysis of the bHLH TFs has been reported in *Brachypodium distachyon*, an emerging model plant in Poaceae.

**Results:**

A total of 146 bHLH TFs were identified in the *Brachypodium distachyon* genome and classified into 24 subfamilies. BdbHLHs in the same subfamily share similar protein motifs and gene structures. Gene duplication events showed a close relationship to rice, maize and sorghum, and segment duplications might play a key role in the expansion of this gene family. The amino acid sequence of the bHLH domains were quite conservative, especially Leu-27 and Leu-54. Based on the predicted binding activities, the BdbHLHs were divided into DNA binding and non-DNA binding types. According to the gene ontology (GO) analysis, BdbHLHs were speculated to function in homodimer or heterodimer manner. By integrating the available high throughput data in public database and results of quantitative RT-PCR, we found the expression profiles of BdbHLHs were different, implying their differentiated functions.

**Conclusion:**

One hundred fourty-six BdbHLHs were identified and their conserved domains, sequence features, phylogenetic relationship, chromosomal distribution, GO annotations, gene structures, gene duplication and expression profiles were investigated. Our findings lay a foundation for further evolutionary and functional elucidation of *BdbHLH* genes.

**Electronic supplementary material:**

The online version of this article (doi:10.1186/s12864-017-4044-4) contains supplementary material, which is available to authorized users.

## Background

Grasses (Poaceae), such as rice, maize, wheat, provide the bulk of nutrition and sustainable energy [1, 2]. Crop growth, development and productivity are continuously threatened by various adverse environmental factors including biotic and abiotic stresses for their sessile nature. They have evolved complicated physiological and biochemical responses by regulating the expression of a series of genes to survive and flourish under extreme living conditions.

Transcription factors (TFs) play key roles in the stress-related regulation network and signal pathways. Among them, basic helix-loop-helixes (bHLH) TFs constitute a large superfamily that has been identified in all eukaryotes including metazoans, plants, and fungi [3–5]. As the second largest class of plant TFs [6], bHLH was characterized with one specific bHLH domain, including a basic region and an HLH region [7]. The basic region, located at the N-terminus of the domain, consisting of approximately 17 amino acids, is a DNA-binding region that enables bHLH TFs to bind to E-box (CANNTG) [7, 8]; the HLH region includes two amphipathic α helices separated by a variable (both in length and primary sequence) loop and participates in the formation of homodimers or heterodimers [8, 9].

In metazoans, the bHLH TFs were divided into six groups (group A to F) based on their phylogenetic relationships, major functions and DNA-binding ability [8, 10–12]. Phylogenic tree of plant bHLHs was first constructed in *Arabidopsis* and AtbHLHs were divided into 12 subfamilies [13]. In rice, the phylogenic tree of bHLH TFs were divided into 22 subfamilies [14]. With more sequenced plant genomes, lots of bHLH proteins were identified. For example, genomes of *Nicotiana tabacum*, *Daucus carota*, *Salvia miltiorrhiza*, and *Solanum lycopersicum* contain 190, 146, 127, 159 *bHLH* genes respectively [15–18]. Researches in *Arabidopsis* revealed that bHLH TFs have versatile biological functions, such as regulating the seed germination [19], the development of epidermal cell [20], carpel [21] and anther [22], fruit dehiscence [23], responding to phytochrome [24] and phytohormone signal [25], stresses [26], etc.

*Brachypodium distachyon*, as the first sequenced species in Pooideae subfamily, has been proposed as a new model organism for functional genomics studies, due to the facility of cultivation and mature transformation system, short life cycle, small genome size and close relation to several cereals [1, 27, 28]. In this study, we identified 146 *BdbHLH* genes and conducted a genome-wide bioinformatics analysis based on the phylogenetic relationships. Meanwhile, the cis-elements in the promoter region, gene structure, conserved motifs, as well as chromosomal distribution, gene duplication and evolutionary mechanisms were investigated. Furthermore, the expression profiles of the BdbHLHs were investigated based on the published RNA-seq, microarray data and qRT-PCR. These results provide clues for functional elucidation of BdbHLHs.

## Methods

### Genome-wide identification, sequence alignment and phylogenetic analyses of *BdbHLH*s

Previous studies indicated that the bHLH domain in *Arabidopsis* contained 19 conserved amino acid residues distributed in the basic region (5), the first helix (5), the loop (1) and the second helix (8) (Additional file 1: Table S1) [29]. Among them, nine mismatches were allowed for the identification of bHLHs [7]. To identify candidate *bHLH* genes in *Brachypodium distachyon*, a BLAST of the bHLH domain was conducted based on the conserved bHLH motif in *Arabidopsis* and rice from the National Center of Biotechnology Information database (http://www.ncbi.nlm.nih.gov) and the Gramene database (http://www.gramene.org/). SMART [30] was applied to verify the candidate bHLH TFs. Proteins with less conserved bHLH domains or no bHLH domains were removed. The biochemical properties were predicted by ExPASy [31]. The GO (gene ontology) annotations of BdbHLHs were obtained from Gramene and Plant Transcriptional Regulatory Map [32], then were visualized by BGI WEGO website [33].

To investigate the phylogenetic relationship between bHLH proteins, protein sequence alignment was performed with default parameters and an un-rooted phylogenetic tree was constructed by MEGA (vision 6.0) [34] based on the neighbor joining (NJ) method with 1000 bootstrap replications and visualized by the EvolView [35].

### Analysis of chromosomal distribution, gene duplication and synteny

The chromosomal distribution of *BdbHLH* genes was obtained from the *Brachypodium distachyon* genome annotations. Tandem duplication events were characterized as contiguous homologous genes on a single chromosome without any intervening gene and checked manually [36]. To analyze the collinear correlations between bHLHs in *Brachypodium distachyon* and rice, maize, sorghum, synteny blocks were downloaded from the Plant Genome Duplication Database [37]. The chromosomal distribution of BdbHLHs and the synteny relationships of related genes across the four species were visualized using Circos (vision 0.69) [38].

### Analyses of promoters, gene structure, conserved motifs, and construction of the interaction network

The upstream 1500 bp genomic DNA sequences of *BdbHLH* genes were downloaded and submitted to the PlantCARE [39] to predict the putative cis-elements. The intron-exon organizations of *BdbHLH*s were displayed by the Gene Structure Display Server [40]. Conserved motifs of BdbHLHs were identified by MEME server [41] with maximum number of motifs set at 15 and optimum width of motifs from 5 to 200 amino acids. The interaction network was constructed based on homologs of BdbHLHs in *Arabidopsis* using the AraNet V2 tool [42] and visualized by Cytoscape (version 3.4.0) [43].

### Analyzing the expression profiles of BdbHLHs

To analyze the expression profiles of BdbHLHs in different tissues and under phytohormone stresses, microarray data (SRP008505) [44] and high throughout RNA sequencing data (PRJDB2997) [45] were retrieved from EBI ArrayExpress (https://www.ebi.ac.uk/gxa/home) and DDBJ Sequence Read Archive (http://www.ddbj.nig.ac.jp/index-e.html) respectively, and then visualized by the MeV (version 4.9.0) [46].

For qRT-PCR, 2-week-old seedlings of *Bd21* were used for different treatments. For salt, drought and plant hormone treatments, seedlings were treated in MS liquid medium containing 200 mM NaCl, 20% PEG6000 (to mimic drought stress), 100 μM MeJA, 100 μM ABA, 20 μM 6-BA and 1 mM SA for 2 h respectively and the roots were collected. For heat and cold treatments, seedlings were subjected to 45 °C and 4 °C respectively and the leaves were collected. Plants during heading stage were used for the collection of roots, stems, leaves and inflorescences. All materials were frozen in liquid nitrogen and stored at −80 °C for RNA isolation. RNA extraction, cDNA synthesis and qRT-PCR reaction were carried out as described previously [47]. The qRT-PCR reaction was performed in triplicate and data acquisition and analyses were performed using the QuantStudio™ Real-Time PCR Software (ThermoFisher Scientific). Samples were normalized using UBC18 (*BRADI4G00660*) expression [48] and relative expression levels were determined using the 2(−ΔΔCt) analysis method [49]. The primers used were listed in Additional file 1: Table S2.

## Results and discussion

### Identification, chromosomal distribution and physicochemical properties of BdbHLHs

With the criterion above, we searched proteins consisting of the conserved bHLH domain in the whole genome of *Brachypodium distachyon*. 146 BdbHLH proteins were identified. The ratio of *bHLH* genes in *Brachypodium distachyon* genome was about 0.55%, which is similar to *Arabidopsis* (0.59%) [29] and is more than rice (0.44%) [14] and poplar (0.40%) [4]. In order to verify the reliability of our identification, a BlastN program was used to search for all the expressed sequence tags (EST) in *Brachypodium distachyon* (Additional file 1: Table S3). 57.5% (84/146) of BdbHLHs were supported by the EST hits.

**Fig. 1:**
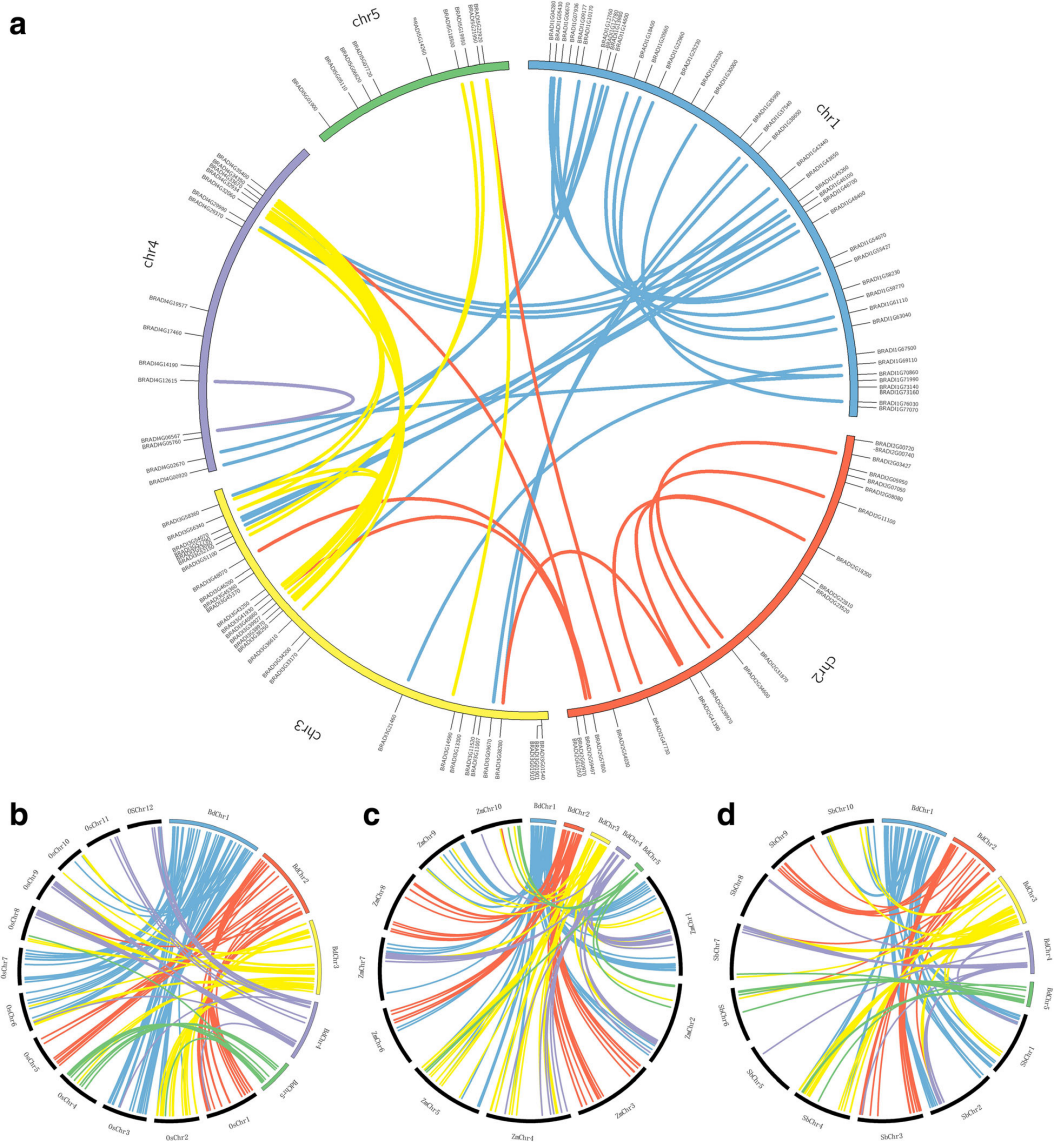
Genomic distribution of bHLH genes and the gene duplications in the *Brachypodium distachyon* (**a**), rice (**b**), maize (**c**), and sorghum (**d**) genome

According to their physical positions (Additional file 1: Table S3), the 146 *BdbHLH* genes were mapped on five chromosomes (Fig. 1a): 47 (32.2%) on chromosome 1, 28 (19.2%) on chromosome 2, 39 (26.7%) on chromosome 3, 21 (14.4%) on chromosome 4, and 11 (7.5%) on chromosome 5. Similar to rice [14], tomato [18, 50] and the common bean [51], most BdbHLHs were found to be located at the both ends of chromosomes 1, 2, 3, 4 and the bottom of chromosome 5.

To further characterize the BdbHLHs, we analyzed the physicochemical properties of the putative proteins (Additional file 1: Table S3). The Grand average of hydropathicity (GRAVY) of all the candidate BdbHLH proteins was predicted to be negative value ranging from −0.026 to −1.003, representing a hydrophilic characteristic. These proteins showed diversities in the length, molecular weight, theoretical isoelectric points (PI), number of negatively charged residues (Asp and Glu), number of positively charged residues (Arg and Lys).

### Gene duplication and collinear correlations of bHLHs between *Brachypodium distachyon* and rice, maize and sorghum genomes

Among the 146 BdbHLHs, about two thirds were duplicated genes. A total of 19 (13.0%) BdbHLHs have been identified as tandem duplicated genes and distributed on chromosome 1, 2 and 3 (Fig. 1a, Additional file 1: Table S4), while *BRADI1G12760* contains an incomplete bHLH domain and might lose the function during duplication [52]. Most of them were derived from the same subfamily with original genes (except for the pair *BRADI3G52790* and *BRADI3G53060* in chromosome 1). By contrast, 75 (58.2%) segmentally duplicated *BdbHLH*s were detected (Fig. 1a, Additional file 1: Table S5)

The substitution rates of non-synonymous versus synonymous (Ka/Ks) is an effective criterion to judge the selection pressure after gene duplications [53]. Thus, the Ka/Ks of duplicated BdbHLHs was calculated (Additional file 1: Tables S4 and S5). For most tandem duplicated gene pairs, the Ka/Ks value was less than 1, indicating a purifying selection during expansion, except for *BRADI2G00730/BRADI2G00740*, *BRADI3G41940/BRADI3G41950* and *BRADI3G52790/BRADI3G52790*, the Ka/Ks ratio was 1.99, 1.63 and 1.15 respectively. This means accelerated evolution is accompanied with positive selection. For the segment duplicated gene pairs, all the Ka/Ks was less than 1 (ranging from 0.14 to 0.82) and the average is 0.46 (Additional file 1: Table S5), suggesting an intense purifying selection pressure during evolution. Meanwhile, the divergence time of the segment duplication event was predicted to take place around 76 Mya, which was much earlier than the tandem duplication (~42 Mya).

To further investigate the origin and evolutionary relationships of *bHLH* genes, comparative syntenic analyses at genome-wide level between *Brachypodium distachyon* and other grass species were conducted. Most BdbHLHs have orthologous in rice, maize and sorghum (80.8%, 69.9% and 72.6%, respectively) (Fig. 1b–d, Additional file 1: Tables S6–S8). The divergence time in rice, maize and sorghum was about 52 Mya, 56 Mya, and 59 Mya, respectively. The Ka/Ks ratio between *Brachypodium distachyon* and rice, maize, sorghum was 0.41, 0.41 and 0.32, implying these *bHLH* gene pairs have gone through strong purifying selection and there was an intimate correlation between *Brachypodium distachyon* and rice, maize, sorghum. In brief, gene duplication events including tandem duplication and segment duplication seemed likely to be essential for *bHLH* gene family expansion and functional conservation and divergence in the Poaceae.

### Multiple sequence alignment, prediction of protein dimerization activity and DNA binding activity of BdbHLHs

**Fig. 2:**
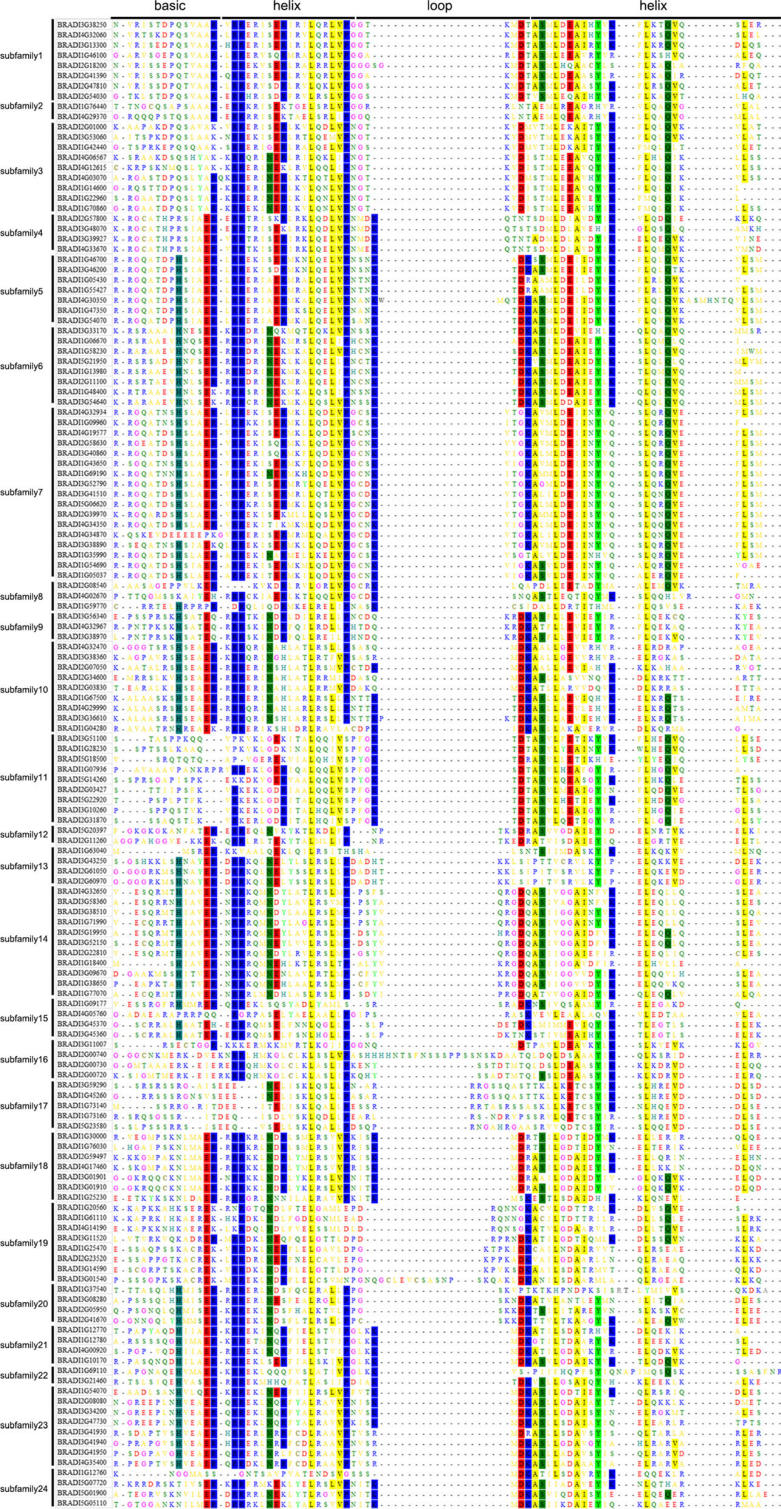
Multiple Sequence Alignment of the bHLH Domains. The amino acids with identity more than 50% are labeled with colored boxes

As shown in Fig. 2 and Additional file 1: Table S1, 24 conserved amino acids were found in the bHLH domains (conservation more than 50%). Among them, Ile-20, Asn-21, Leu-24, Gln-28, Lys-36, Asp-38, Ile-43, Val-51 and Leu-54 were more conservative in plants [4, 54]. Some conserved amino acid residues such as Glu-13, Arg-14, Arg-16 and Leu-27 were not only detected in *Brachypodium distachyon*, but also in *Arabidopsis* and rice, suggesting they are essential to the biological function of bHLH proteins [3].

**Fig. 3:**
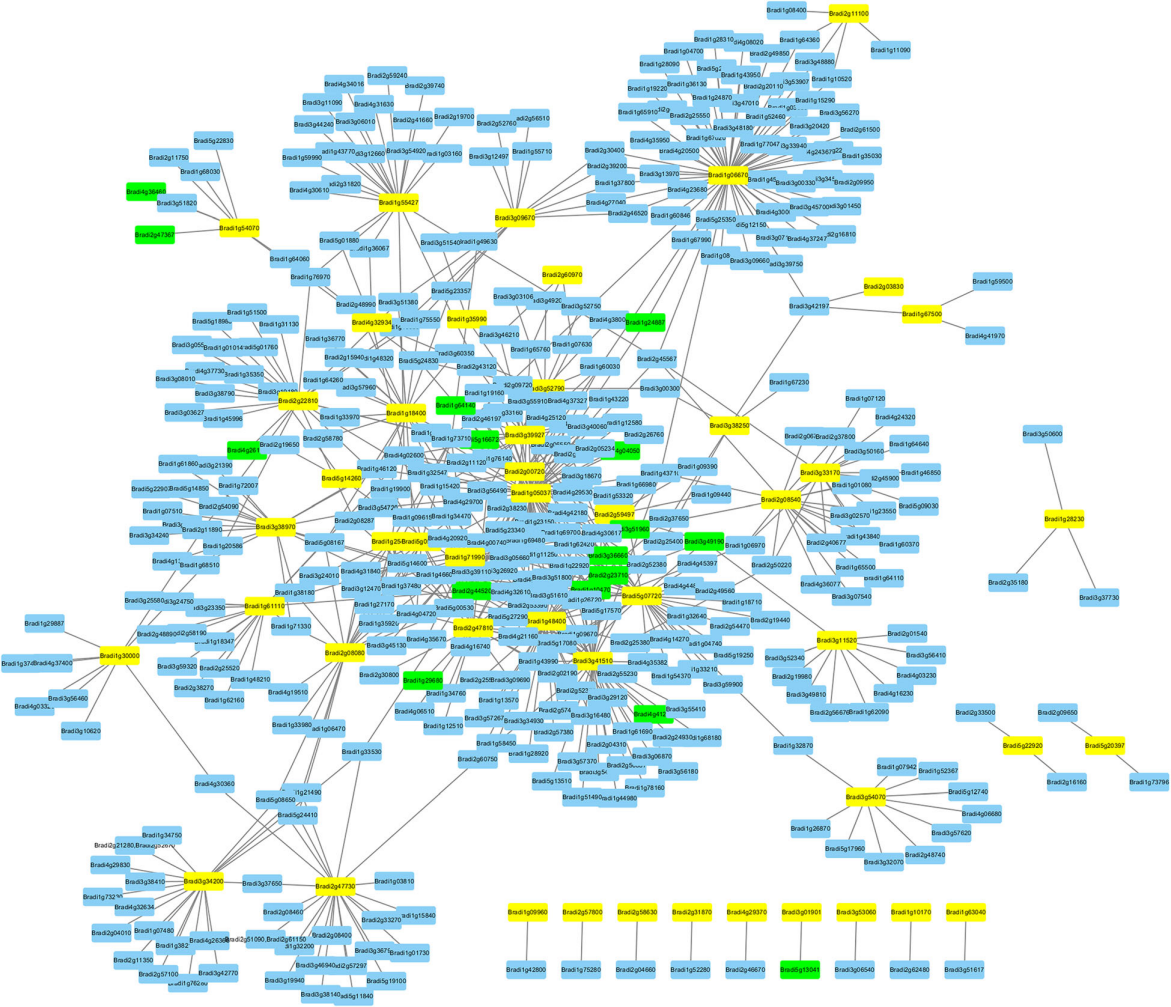
The interaction network of bHLHs in *Brachypodium distachyon* according to the orthologs in *Arabidopsis*. The BdbHLHs are in yellow blocks, the MYBs are in green blocks while other genes are in blue blocks

Previous studies indicated that the HLH domain was essential in both dimerization and DNA binding [9]. Especially, Leu-27 in helix 1 and Leu-73 in helix 2 were important for protein interaction [4]. In this study, 145 and 144 BdbHLHs were found to have Leu-27 and Leu-54 (corresponding to Leu-73 in AtbHLHs) respectively (Fig. 2). Recently, MYC2, MYC3 and MYC4 in *Arabidopsis* were reported to form homodimers through Leu, Ile and Val in the helixes [55]. In *Brachypodium distachyon*, including three homologues of AtMYC2, AtMYC3 and AtMYC4, the helixes in many BdbHLHs have these three kinds of amino acids simultaneously, implying the probability to form protein complexes (Fig. 2). In combination with GO annotation that all BdbHLHs showed protein dimerization activity (GO: 0046983, Additional file 1: Tables S9, S10 and Additional file 2: Figure S1), we speculated that BdbHLHs might function by forming protein complex and tried to construct the interaction network of BdbHLHs. Because of short of reported experiment data and databases, the interaction network was constructed based on the orthology analysis with AtbHLHs. According to the AraNet V2 [42], 57 BdbHLH proteins have orthologs in *Arabidopsis*. As a result, 660 interaction protein pairs were predicted (Fig. 3, Additional file 1: Table S11). It has been reported that members of bHLHs and MYB gene families might function cooperatively via physical interaction [6, 56–58], so the interaction network was further analyzed and a total of 16 MYB genes based on PlantTFDB database [59] were sought out (Fig. 3). The interaction network might provide some clues to investigate the molecular mechanism of bHLH.

**Fig. 4:**
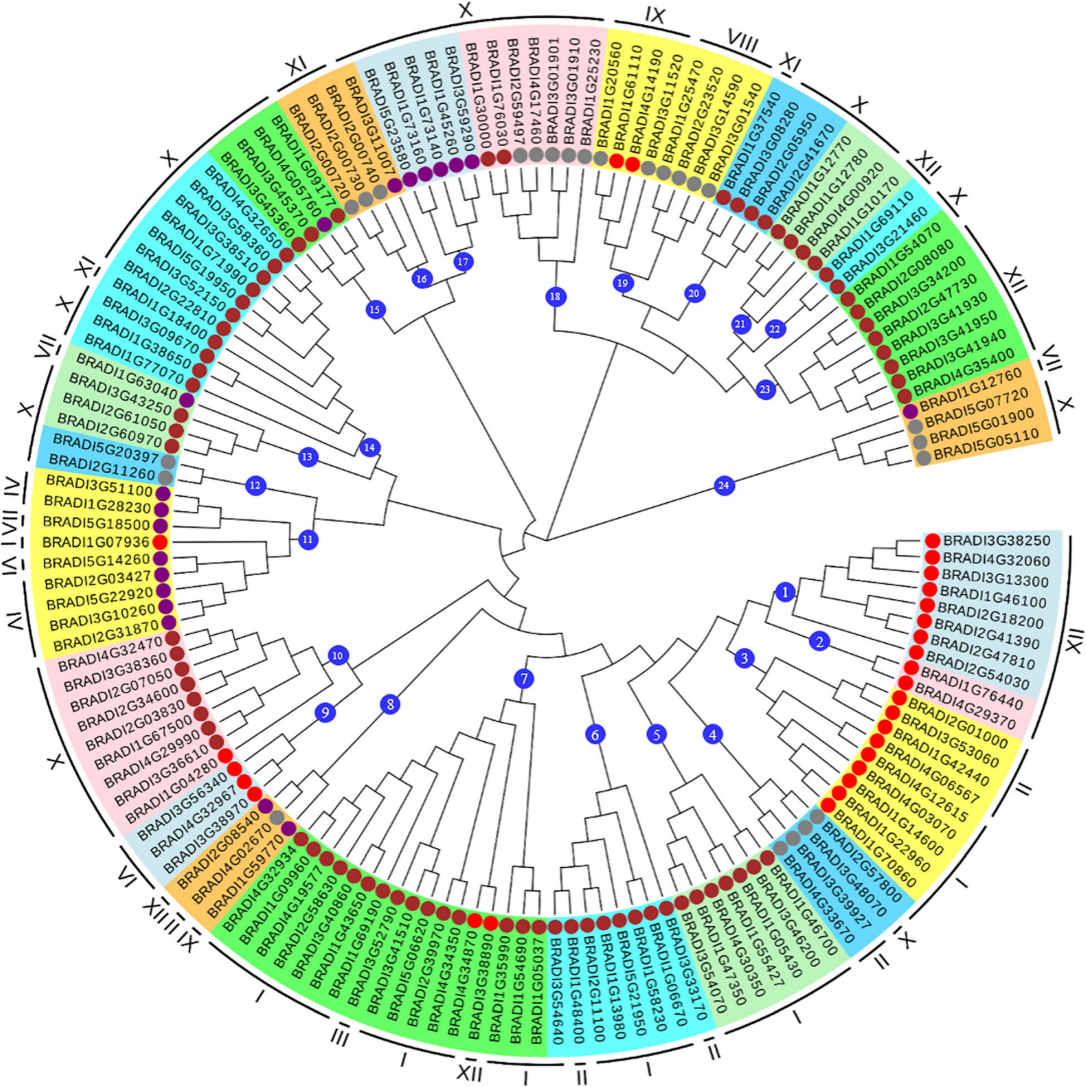
Phylogenetic tree constructed with the BdbHLH domains based on the neighbor-joining method. The tree shows the 24 phylogenetic groups (numbered within blue circles). The circles of different colors represent the predicted DNA-binding activity of each protein: G-box in brown, Non-G-box in gray, Non-E-box in red and No-DNA binding in purple

The BdbHLHs were grouped based on the amino acid sequence of the bHLH domain which determines the DNA binding activity [29]. Glu-13 is related to CA in the E-box DNA binding motif (CANNTG) and the substitution of it with other residues (Gln, Asp and Leu) abolishes the DNA binding activity [60–64]. Arg-16 could stabilize the position of Glu-13 and was essential in DNA binding [61, 62]. Based on the existence of Glu-13 and Arg-16 which play a key role in recognition of E-box, the BdbHLH proteins were divided into E-box binding and non-E-box binding (Additional file 1: Table S12) [62, 65]. In addition, His/Lys-9, Glu-13, and Arg-17 are responsible for the specificity to bind to G-box (CACGTG). Especially, Arg-17 directly interacts with the middle G and His-9 interacts with both the middle G and the first C of G-box [62, 63, 65]. So, the E-box-binding proteins were further divided into G-box-binding proteins and non-G-box binding proteins according to the presence of His/Lys-9, Glu-13 and Arg-17 residues or not. For example, it was reported that the G-box binding protein AtMYC2 was crystallized in complex with G-box DNA. Similar to mammalian bHLH TFs, further research showed that three conserved amino acids H453, E457 and R461 (corresponding to our His-9, Glu-13 and Arg-17, respectively, Fig. 2) were essential for the formation of the complex [55]. Meanwhile, the MYC2-DNA structures can further form homo-tetramer with significantly enhanced DNA binding affinity due to the interaction between conserved R458, Q459 and Q466 in one dimer with DNA in the other dimer [55]. In *Brachypodium distachyon*, three bHLHs, BRADI2G08080, BRADI3G34200 and BRADI2G47730 are highly conservative with AtMYC2 and possess Arg-14, Gln-15 and Gln-22 amino acids (corresponding to R458, Q459 and Q466, respectively), suggesting that they could form tetramers too. The bHLHs without predicting E-box-binding specific recognition residues but possessing additional basic amino acids in the basic region might be able to bind DNA without specificity for E-boxes were classified as non E-box DNA binders [62, 65]. According to the conservation of these residues, 102 BdbHLH proteins were predicted to be putative E-box-binding proteins wherein 78 belong to G-box-binding proteins, 25 as non-E-box-binding proteins for missing Glu-13/Arg-16 residues while 19 BdbHLHs containing less than six amino acid residues in the basic region fell into non-DNA-binding proteins (Fig. 4). Non-DNA-binding proteins, also known as HLH protein, might function like MYB-bHLH-WD40 which can interact with DNA binding proteins as negative regulators [66].

### Gene structures, conserved motifs and functional prediction of BdbHLHs based on phylogenic analyses

A Neighbor Joining phylogenetic tree was constructed based on the bHLH domains of the BdbHLHs (Fig. 4). According to the values obtained in the bootstrap analysis, the bHLH domain in the outer clades had better resolution, permitting subfamilies of proteins to be delimited. The results showed that the BdbHLH amino acid sequences in the same subfamily were highly conserved, implying a strong evolutionary relationship among those members. Based on the statistical support of each branch, we selected those with a bootstrap value >50 to divide the BdbHLH proteins into 24 subfamilies. According to previous phylogenic classifications [4, 13], another phylogenic tree was constructed based on the full length alignments of the 146 BdbHLHs, 167 AtbHLHs and 39 functionally annotated OsbHLHs (Additional file 1: Table S13, Additional file 2: Figure S2). According to the evolutionary relationship, thirteen major subfamilies in *Arabidopsis* and rice were classified in consistent with the BdbHLH phylogenic tree except for subfamily VI and XIV (Additional file 2, Fig. S2) [4, 13].

**Fig. 5:**
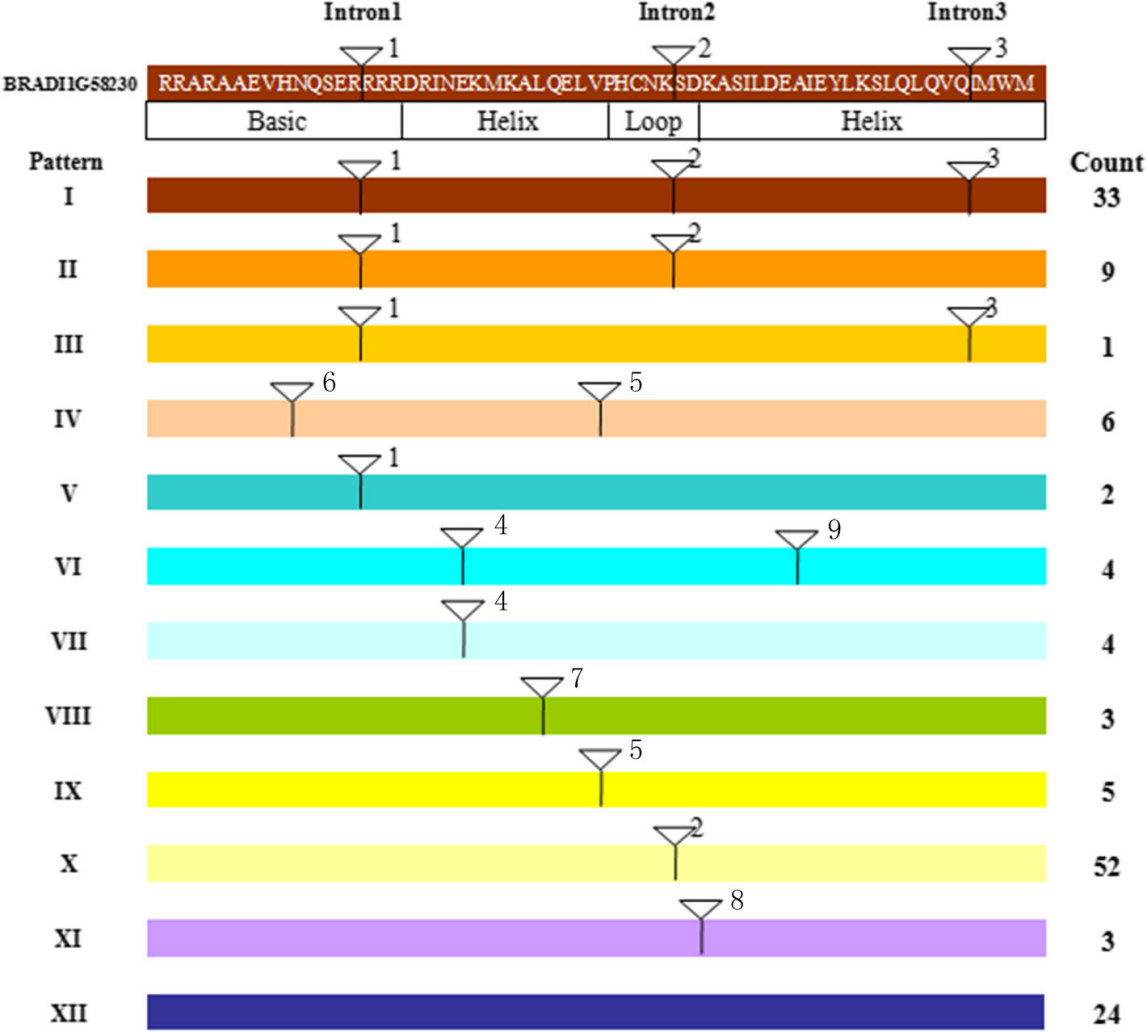
Intron distribution patterns in the coding sequence of the bHLH domain of BdbHLHs. The intron distribution patterns are marked with different colors, and position of introns is indicated by triangles. BRADI1G58230, as an example, is shown at top

Exon/intron organization, as a type of structural divergence, plays an important role in the evolution of gene families [67]. As shown in Fig. 5, 122 were found to possess introns in their bHLH domains among the 146 BdbHLH proteins. 9 conserved intron positions and 12 different intron distribution patterns (I ~ XII) were identified in our study and pattern IV (introns position in His-9 and Val-31) with 6 members was not present in *Arabidopsis* [26]. BdbHLHs possessing diverse gene structures in the conserved bHLH domains were regularly distributed in the phylogenic tree (Fig. 4). For example, the majority of pattern I and X members were found in the subfamilies 4–7 and 12–16 respectively. In contrast, the members in the subfamilies 11 and 17, which were almost the Non-DNA binding type proteins, had pattern IV and pattern X respectively.

Additionally, members of the same subfamily also displayed similar intron distribution patterns in view of the full-length genome sequences (Additional file 2: Fig. S3). For instance, all the BdbHLHs in subfamily 1 had only one exon, the whole members of subfamily 10 showed 2 exons while 7 out of 9 members in the group3 consisted of 5 exons which were in similar length and at similar positions.

In addition to bHLH domain, bHLH proteins in different subfamilies have different additional motifs which might be formed during evolution [7]. Totally, 15 conserved motifs were found (Additional file 2: Figure S3). Motif 1 and motif 2, located in bHLH domains, were found in almost all BdbHLHs (except for the BRADI4G05760 and BRADI1G12760). It is noteworthy that the BdbHLHs in the same subfamily were more likely to share same motif and location, which might imply similar biological functions [68]. For instance, a KRAAM motif before bHLH domain, which was reported to be involved in cold acclimation response [69], was found in BRADI2G59497 and BRADI4G17460 in subfamily18.

In *Arabidopsis* and rice, functions of many bHLH proteins have been characterized. In the subgroup Ia, MUTE [70], FMA [71, 72], SPCH [73–75], bHLH071 [71] associated with stomatal development and HWS [76] was related to sepal fusion and organ size. Meanwhile, MUTE, FMA and SPCH2 in rice were also reported to play a role in the differentiation and development of stomatal cells [77], implying that members in the subgroup Ia might be mainly involved in cell division and organ differentiation. In subgroup Ib, ORG2, ORG3 [78–81], bHLH100 and bHLH101 in *Arabidopsis* [80–83] play a key role in response to iron-deficiency. Additionally, OsIRO2 is also an essential regulator of Fe uptake and iron utilization in rice [84–86]. One except is, AtRGE1 primarily functions in embryo growth [87–91]. In *Arabidopsis*, three subgroup II members bHLH010, bHLH089 and bHLH091 were reported to interact with DYT1 proteins and redundantly participate in the anther development [92]. Similarly, rice subgroup II members EAT1 and TIP2 play a key role in the anther development at early stage [93, 94]. In subgroup IIIa, AMS [95–98] and DYT1 were identified to be master regulators of pollen development [99, 100]. Consistently, rice UDT1 interacted with TDR to regulate early anther development [101–105]. AtICE1, AtICE2 and OsICE1, OsICE2 in IIIb were proved to participate in the response to deep freezing [69, 106–110] while another member NFL was involved in GA mediated control of flowering time [111]. In *Arabidopsis*, only one member of subgroup IIIc, At4g29930, was functionally characterized. It might regulate hypocotyl and root elongation [112] while one member of IIIc in rice showed a correlation to JA inducible transcriptional activation during wound and drought stresses [113]. In *Arabidopsis*, IIId members (JAM1-JAM3 and bHLH014) and IIIe bHLHs (MYC2-MYC4) were proposed to take part in JA-mediated plant development [114–119]. In rice, OsMYL1 and OsMYL2 interact with OsMYC2 to participate in the JA signalings [120–122]. Subgroup IIIf protein TT8 participates in anthocyanin and PA pathways [123], similar to IIIf members in rice that involved in anthocyanidin biosynthesis [124–126]. Besides, three other IIIf proteins in *Arabidopsis* were found to be involved in the development of epidermal cells (GL3, EGL3 and MYC1) [127–130]. IVb (PYE) and IVc proteins (bHLH034, bHLH104, bHLH115 and ILR3) were proved to modulate metal homeostasis [26, 131–133]. Similarly, rice OsIRO3 of subgroup IVb also regulates iron homeostasis [134]. In IVd, *AtbHLH092* responds to osmotic stress and regulates circadian rhythms [135, 136]; *OsDPF* participates in the resistance to diseases [137]. Va members (BIM1, BIM2 and BIM3) were suggested to participate in brassinosteroid signal and positively modulate the shade avoidance syndrome in seedlings [138, 139] while Vb members showed regulatory capacity in diverse processes including vascular development (ABS5 and TMO) [140, 141], hypocotyl and root elongation (At2g40200) [112] and responses to abiotic stresses (STC8) [142]. MEE8 in subgroup VI was speculated to regulate genes necessary to embryo and endosperm biogenesis [143]. The reported VIIa members (PIF1, PIF3-PIF5, PIL1 and PIL2) showed a relationship with photo induced signal transduction [144–155]. The rice subgroup VII proteins (OsPIL1, OsPIL11, OsPIL12, OsPIL14, OsPIL15 and APG) were functional counter-parts of PILs in *Arabidopsis* and involved in red light-mediated signal transduction pathways [156–161]. In addition, VIIb members in *Arabidopsis* are involved in cotyledon expansion and regulated seed dormancy (SPT) [162, 163], cell separation in fruit dehiscence (ALC) and interact with phytochromes (UNE10, RSF1 and PIF7) [164–167]. Two subgroup VIIIa members PAR1 and PAR2 were reported to integrate hormone and shade transcriptional networks and redundantly function in the enhancement of seedling de-etiolation related to phytoreceptor signal [139, 154, 168–171]. In subgroup VIIIb, HEC1-HEC3 modulated the development of the transmitting tract and stigma [172], fruit opening (IND) [173] and axillary meristem formation (ROX) [174]. Three rice VIIIb proteins have been studied, i.e. OsbHLH120 might control root thickness and length [175]; OsLF negatively regulated flowering [159, 176] and LAX specified the terminal spikelet meristem [177–179]. Members in subgroup VIIIc (RHD6, RSL1, RSL2, RSL4 and At2g14760) were verified to be essential to root hair development [130, 180, 181], while OsbHLH133, one characterized subgroup VIIIc member in rice, was proved to regulate the iron distribution between root and shoot [182]. Subgroup IX bHLHs were proposed to be involved in photoperiodism flowering (FBH1-FBH4) [183] and facilitate stomatal opening through phosphorylation (AKS2) [184]. Two studied X proteins (bHLH068 and bHLH112) could response to abiotic stresses [185, 186]. Subgroup XI members in *Arabidopsis* (LRL1-LRL5) [130, 187, 188] and rice (OsRHL1) [189] regulate root hair development, while another protein OsPTF1 is involved in phosphate starvation tolerance [190]. Subgroup XII members displayed diverse regulation functions: to monitor brassinosteroid signaling, to respond to freezing tolerance (CESTA, BEE1-BEE3 and BHI1) [191–195] and to promote flower initiation and regulate cell elongation (CIL1, CIL2 and CIB1-CIB5) [196–198]. In rice, only one XII member, An-1, has been functionally characterized. It regulates awn development, grain size and grain number [199]. Subgroup XIII proteins (UPB1, LL1, LL2 and LHW) were mainly required for the establishment and maintenance of normal vascular differentiation and development [200–203]. Subgroup XIV members (SAC51 and SACL1–3) might be involved in the response to thermospermine and xylem differentiation [204–206]. Subfamily XV members (PRE1–6) tend to be take parts in light, brassinosteroid and gibberellin signaling and modulation of flowering time [207–211]. In rice, four proteins of subgroup XV were reported to be involved in brassinosteroid signal (ILI1 and BU1) [212, 213] and regulation of grain length and weight (PGL2) [214].

The analysis of the functional characterized bHLH proteins in different subgroups in *Arabidopsis* and rice above, indicate the conservative function of bHLH members from different species in the same subgroups. According to the phylogenic tree, 79 BdbHLHs distributed in 21 subfamilies were found to have functionally characterized homologous proteins in *Arabidopsis* and rice (Additional file 1: Table S13). Based on these results, the functions of these 79 BdbHLH TFs could be predicted to some extent. Researches on several functionally characterized BdbHLH proteins further support this opinion.

One example is, in *Arabidopsis*, two RSL class I proteins AtRHD6 (ROOT HAIR DEFECTIVE6, AtbHLH083) and AtRSL1 (ROOT HAIR DEFECTIVE 6-LIKE 1, AtbHLH086), were reported to regulate the expression of the RSL2 and RSL4 and function as positive regulators to regulate the development of root hair cells [180, 215–217]. In our phylogenic tree, BRADI2G01000, BRADI3G53060 and BRADI1G42440 and AtRHD6, AtRSL1 were tightly grouped within a subfamily, indicating high homology among them. As predicted, BdRSL1(BRADI2G01000), BdRSL2 (BRADI3G53060) and BdRSL3 (BRADI1G42440) do function in the development of root hair cells [180, 218].

The other example is, in *Arabidopsis*, group IIIb members AtICE1 (AtbHLH116) and AtSCRM (AtbHLH033) function together with group Ia member AtSPCH (AtbHLH098) to regulate stomatal development [106]. BdICE1 (BRADI4G17460) and BdSCRM2 (BRADI2G59497) were tightly grouped with AtICE1 and AtSCRM in subfamily 18 in our phylogenic tree. Consistently, BdICE1 and BdSCRM2 cooperated with BdSPCH1 (BRADI1G38650) and BdSPCH2 (BRADI3G09670) to regulate the stomatal development despite the differences of their individual roles [219].

### Expression profiles of *BdbHLH*s

**Fig. 6:**
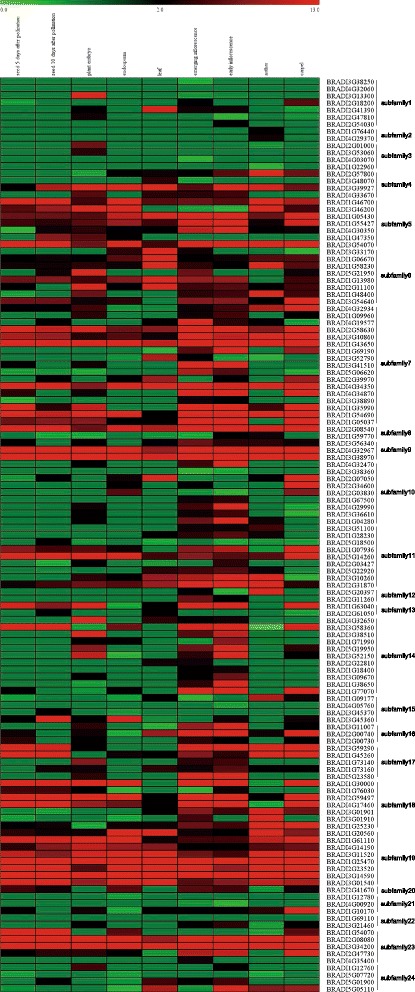
The expression profiles of *BdbHLH* genes in different tissues. The color scale is shown at the top. Higher expression levels are shown in red

Since the functions of genes associated with their expression patterns, the expression profiles of *BdbHLH* genes were analyzed. According to the available microarray data, the expression levels of *BdbHLH* genes in 9 different tissues varied considerably (Fig. 6). Genes in the same subfamily showed similar expression profiles at some level. For example, majority of genes in subfamily 4, 5, 6, 7, 8, 9, 18 and 19 showed relatively high expression in all detected tissues while the bulk of genes from subfamily 1, 2, 3, 10 and 21 showed lower or no expression. In contrast, some subfamilies were found to be specifically expressed in certain tissues. For example, genes from subfamily 2 were specifically expressed in anther; genes from subfamily 12 were found only in inflorescences and anther; the expression of subfamily 14 members were mainly found in plant embryo, emerging inflorescences and early inflorescences, implying that their functions have been differentiated.

**Fig. 7:**
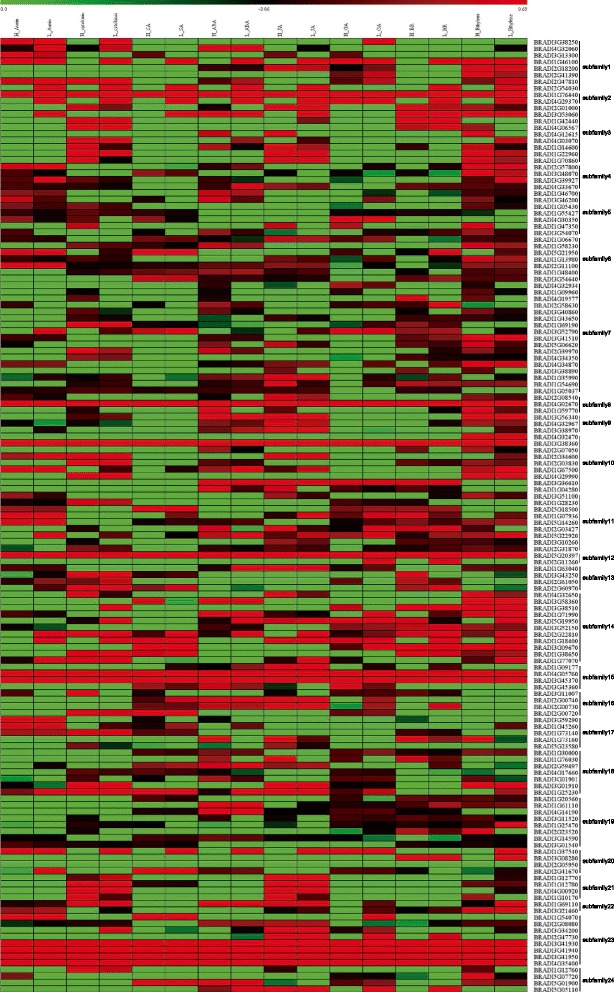
The expression profiles of BdbHLHs treated with high and low concentration of various phytohormones. The heatmap was generated with log2 based values. The color scale is shown at the top. Higher expression levels are shown in red while lower expression levels are shown in green. H stands for high concentration of phytohormone treatment while L stands for low concentration treatment

Meanwhile, the expression level of different BdbHLHs was up or down-regulated by different phytohormones (Fig. 7). Genes in the same subfamily showed similar responses to exogenous phytohormones to some extent. For example, auxin down-regulated the expression of genes from subfamily 3 (except for *BRADI3G53060* which was up-regulated by low concentration), 18 and 21 while high concentration of auxin up-regulated the expression of genes from subfamily 4; cytokine down-regulated the expression of genes in subfamily 20 while up-regulated the expression of genes in subfamily 21; SA down-regulated the expression of most genes in subfamily 9, 22, 3 (except for *BRADI3G53060* which was up-regulated by low concentration) and subfamily 5 (at low concentration); ABA up-regulated the expression of subfamily 9 genes and down-regulated the expression of genes from subfamily 21 and subfamily 17 (except for that *BRADI1G45260* was up-regulated by low concentration); JA down-regulated the expression of genes from subfamily 19, 20 and 5 (except for *BRADI1G47350* was up-regulated at high concentration) while up-regulated the expression of genes from subfamily 9 and 21; GA down-regulated the expression of genes from subfamily 3, 9, 17 and 21; brassinosteroid down-regulated the expression of genes from subfamily 1 (low concentration) and subfamily 21; ethylene up-regulated the expression of genes from subfamily 4, 6 (except for *BRADI5G21950* which was down-regulated by high concentration), 8 and 14 (low concentration) while down-regulated the expression of genes from subfamily16.

**Fig. 8:**
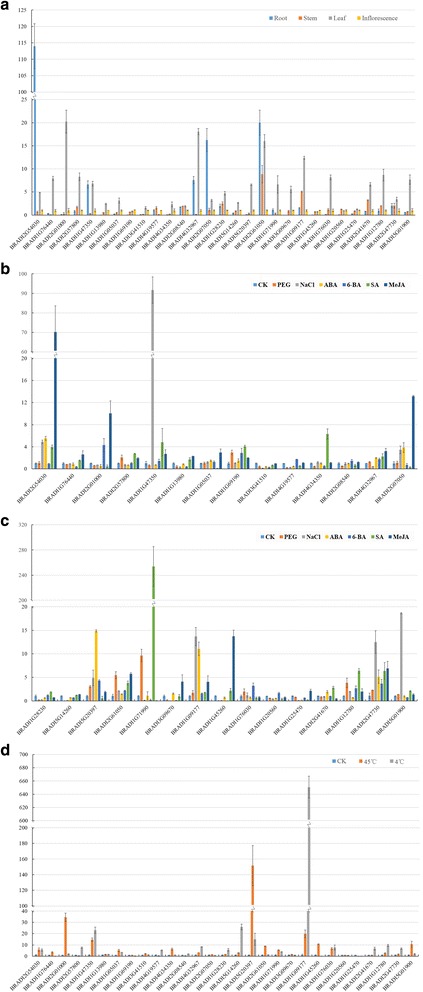
Quantitative RT-PCR analysis of 29 selected *BdbHLH* genes. The relative expression levels of the 29 genes in (**a**) different organs namely root, stem, leaf and inflorescence; (**b** and **c**) root with different treatments including 20% PEG6000, 200 mM NaCl, 100 μM MeJA, 100 μM ABA, 20 μM 6-BA and 1 mM SA; (**d**) leaf with treatments of high temperature (45 C) and low temperature (4 C)

To further investigate the possible expression patterns of bHLHs in different organs and under abiotic stresses, the expression levels of 29 BdbHLHs randomly selected from 22 subfamilies were investigated using quantitative RT-PCR. As shown in Fig. 8a, during heading stage, 29 genes showed different expression patterns. For instance, *BRADI2G54030* and *BRADI2G07050* were primarily expressed in roots, while *BRADI2G01000* showed a preferential expression in leaves. *BRADI2G61050* was expressed high in root, stem and leaves but low in inflorescences. Genes from same subfamily probably display similar expression patterns. *BRADI1G05037*, *BRADI3G41510*, *BRADI4G34350* and *BRADI1G69190* from subfamily 7 showed low expression in four tested tissues and a relatively high expression level in leaves and inflorescences; *BRADI1G28230* and *BRADI5G14260* from subfamily 11 and *BRADI1G71990* and *BRADI3G09670* from subfamily 14 exhibited high expression in leaves while *BRADI1G20560* and *BRADI1G25470* from subfamily 19 were hardly detected in root. Taking the conserved motifs and similar gene structure into account, we predict genes in the same subfamily might play redundant roles to some extent.

Additionally, the expression profiles of 29 selected genes under different abiotic stresses were investigated too (Fig. 8b–d) and the cis-regulatory elements were analyzed to verify our results (Additional file 1: Table S14). In general, the expression patterns are consistent with the analysis of promoters. For example, *BRADI1G47350* and *BRADI2G54030* were strongly induced by NaCl and MeJA, respectively, consistent with the prediction that *BRADI1G47350* contains 5 ABRE cis-elements (element respond to salt stress) and *BRADI2G54030* contains 3 TGACG-motifs (cis-acting regulatory element involved in the MeJA-responsiveness) [220]; *BRADI1G71990* containing 1 TCA-element (cis-acting element involved in salicylic acid responsiveness) was drastically up-regulated by SA. It might interact with *BRADI3G51960* to respond to salicylic acid according to the interaction network. The expression of *BRADI1G09177, BRADI5G01900* and *BRADI5G20397* were up-regulated by NaCl and ABA, consistent with the presence of ABRE cis-elements in these genes. *BRADI1G45260*, *BRADI2G01000* and *BRADI2G07050*, containing TGACG-motifs, showed higher expression under MeJA treatment. For extreme temperature including heat (45 °C) and cold (4 °C), the expression of *BRADI1G09177* and *BRADI5G20397* were strongly up-regulated by cold and heat treatment respectively, in accordance with the presence of DRE (regulatory element involved in cold- and dehydration-responsiveness) [221], or HSE (cis-acting element involved in heat stress responsiveness) [222].

## Conclusions

To study the bHLH gene family in the *Brachypodium distachyon*, an emerging model plant in grass, we identified 146 *bHLH* genes distributed in 5 chromosomes. Gene duplications showed that duplication events, especially segment duplications made up a large proportion in the expansion of BdbHLHs. Synteny analyses indicated that bHLHs in *Brachypodium distachyon* had close relationships with rice, maize and sorghum. GO analysis showed that the majority of BdbHLHs were involved in transcriptional regulation and displayed protein binding ability, suggesting that they might function in homodimer or heterodimer manners. According to phylogenetic analysis of the bHLH domains and the alignment with full-length sequences of *Arabidopsis* and rice, BdbHLH TFs were classified into 24 subfamilies. Based on the functional characterization of homologous genes in *Arabidopsis* and rice, the BdbHLHs were predicted to take part in various processes including growth and development, stress responses and so on. The expression profiles of *BdbHLH* genes in different tissues and under different phytohormones treatments were analyzed, and some tissue-specific and phytohormone-responsive genes were identified. Taken together, our results provide a solid foundation for further evolutionary and functional investigations on BdbHLHs.

## Additional files

Additional file 1: Table S1. Consensus motifs of bHLH domains. **Table S2.** The primers used in the qRT-PCR. **Table S3.** Characteristic features of bHLH Transcription factor gene family identified in *Brachypodium distachyon*. **Table S4.** The Ka/Ks ratios and estimated divergence time for tandemly duplicated *BdbHLH* genes. **Table S5.** The Ka/Ks ratios and estimated divergence time for segmentally duplicated *BdbHLH* genes. **Table S6.** The Ka/Ks ratios and estimated divergence time for orthologous *bHLH* genes between *Brachypodium distachyon* and rice. **Table S7.** The Ka/Ks ratios and estimated divergence time for orthologous *bHLH* genes between *Brachypodium distachyon* and maize. **Table S8.** The Ka/Ks ratios and estimated divergence time for orthologous *bHLH* genes between *Brachypodium distachyon* and sorghum. **Table S9.** GO annotations of BdbHLHs. **Table S10.** GO descriptions of BdbHLHs. **Table S11.** Detailed information of interaction network of BdbHLH with other proteins. **Table S12.** Predicted DNA Binding Characteristics of the bHLH Domain in BdbHLH Proteins. **Table S13.** Summary of the predicted functions of BdbHLHs by comparative analysis with homologous bHLHs in *Arabidopsis* and rice. **Table S14.** The cis-regulatory elements in the promoter region of 29 *BdbHLH* genes. (XLSX 237 kb)

Additional file 2: Figure S1. GO annotations of BdbHLHs. **Figure S2.** Phylogenic tree using alignments of bHLH proteins in *Brachypodium distachyon*, *Arabidopsis* and rice. **Figure S3.** Conserved elements and gene structures of BdbHLHs. (RAR 22526 kb)

Additional file 3: The sequences of all the *BdbHLH* genes, including CDS, amino acids of the bHLH domains, protein and genomic DNA as well as 1500 bp upstream sequences. (RAR 398 kb)

## Abbreviations

ABA: Abscisic acid
bHLH: basic helix-loop-helix
EST: Expressed sequence tag
FPKM: Fragments kilobase of exon model per million mapped reads
GO: Gene ontology
GRAVY: Grand average of hydropathicity
Ka: Substitution rate of non-synonymous
Ks: Substitution rate of synonymous
MeJA: Methyl jasmonate
NJ: Neighbor joining
PI: Isoelectric point
SA: Salicylic acid
TF: Transcription factor
